# Reliability and Validity of the “Personal Well-Being Index – Adult” in Mothers of Mentally Retarded Students in North of Tehran-Iran

**Published:** 2011

**Authors:** Alireza Agha Yousefi, Ahmad Alipour, Nasim Sharif

**Affiliations:** 1 Department of Psychology, Faculty of Humanities, Payam-e- Noor University, Qom, Iran.; 2 Department of Psychology, Faculty of Humanities, Payam-e- Noor University, Tehran, Iran.

**Keywords:** Mentally retarded student, Mothers, Personal Well-being, Reliability, Validity

## Abstract

**Objective:** Having a good quality of life has always been desirable for humans and the concept of a good life and the ways of achieving it have been focused over the years. Personal wellbeing is the mental component of quality of life. Thus, the current study was conducted to estimate psychometric characteristics of “personal well-being index-adult” (PWI-A) in mothers of mentally retarded students of exceptional schools in northern districts of Tehran.

**Methods:** From 358 mothers of mentally retarded students in north of Tehran (districts 1, 2 and 3), 200 mothers were selected by systematic random sampling. The collected data using PWI-A was analyzed by inferential statistics (Cronbach’s alpha coefficient for test reliability and linear multivariate regression for test validity).

**Results:** Results showed acceptable reliability and validity for PWI-A in mothers of mentally retarded students of exceptional schools in north districts of Tehran. The Correlation between seven items was significant (P<0.001) and this index had the most extensive coverage of construct validity.

**Conclusion:** This study showed that this scale could be applied to measure personal wellbeing in mothers of mentally retarded students.

## Introduction

Family adaptation may vary depending on the type of communication used in each family to solve a problem (1). Families with an affirming style of problem-solving communication (that conveys support and care and exerts a calming influence) adapt more successfully to stressful situations than families with an incendiary style of problem-solving communication (that is inflammatory in nature and tends to exacerbate a stressful situation). Well-being is likely to be higher in families that use numerous strategies to solve problems and cope with difficulties. In addition, there is likely to be a positive association between an affirming style of problem-solving and well-being. As noted previously, relatively few researchers have assessed well-being in families of disabled children. Besides, the existing research has mainly focused on negative indicators of well-being. Moreover, the available findings are contradictory and inconclusive. For instance, in a longitudinal study conducted by Carr (2), siblings of children with Down syndrome were reported to have fewer behavioral problems in comparison with others. On the other hand, Gath and Gumley (3) found no difference in behavioral problems between siblings of children with Down syndrome and compared siblings. In a study by McHale and Gamble (4), siblings of children with disabilities showed higher depression, higher anxiety, and lower perceived competence than compared siblings. Lobato et al (5) found that brothers of children with disabilities had significantly higher depression and aggression scores than sisters of children with disabilities or siblings in the comparison group. Additionally, sisters of children with disabilities had higher aggression scores than comparison sisters. No difference was found between the perceived competence of siblings of children with disabilities and comparison siblings.

Personal well-being (PWB) is the most global term used to describe how people feel about their lives. It includes people’s emotional responses, satisfaction with life domains, and global judgment of life quality (6). Thus, PWB comprises measures of cognition (satisfaction) and affects (positive affect) (7). The cognitive component of PWB can be described in two ways: life satisfaction and subjective quality of life.

Subjective well-being (SWB) has been defined as a construct that reflects peoples’ perceptions of their lives in terms of emotional behavior and psychosocial functioning, which are all essential dimensions of mental health (8). SWB research indicates the domains of personality, motivation and a group of cognitive buffers (perceived control, self-esteem and optimism); all contribute to people’s life satisfaction (9).

PWB is an integral factor of SWB. Studies have found seven domains important to PWB; standard of living, health, achievement in life, safety, relationships, community connectedness, and future security (10). These domains are valuable in understanding of the psychological and physical makeup of individuals and identify components that contribute to PWB generally.

Cummins et al (11) proposed that the body has an internal system which monitors and works to maintain relatively constant levels of PWB. It has been proposed that a diverse range of cognitive strategies and personality factors are responsible for returning well-being to the baseline levels following an overly adverse or positive experience including neuroticism and extroversion (12), optimism (13,14), self-esteem (15) and perceived control (16). Besides, many studies suggest people who have higher levels of PWB may volunteer more often (17).

In another research, Cohen et al. (18) found that people who consider themselves to be connected and close to their neighborhood report high levels of personal well-being.

Miller (19) concluded that the perceived overeating was a better predictor of PWB level than the actual weight. Another study of the effects of perceived eating control and body mass index (BMI) on PWB supported an eating-PWB relationship (20).

It is generally agreed that SWB may be measured though questions of satisfaction focused to people’s feelings about themselves. Personal Well-being Index-Adult (PWI-A) is one of the most famous questionnaires in this area.

Several researches have been done about evaluating the reliability and validity of PWI-A. Sixteen surveys of the Australian population have produced a maximum variation of 3.2 percentage points in PWI-A (see Australian Unity Wellbeing Index Report 16.0) (21).

Furthermore, PWI-A scale has been shown to have a good internal reliability with a Cronbach’s alpha of 0.89 in another research (22). Miller (19) has reported a corresponding coefficient of 0.86. Agha Yousefi (23) conducted a research to evaluate the reliability and validity of PWI-A in the wives of self-sacrificed men in Qom city. According to the results of this research, the reliability of this scale was equal to 0.845 by Cronbach’s alpha coefficient. Davey (24) reported an alpha of 0.87 for PWI-A scale.

Construct and convergent validity of PWI-A have been approved in previous studies*.* A correlation of 0.78 with the satisfaction with life scale has been reported for construct validity (25,26). Regarding convergent validity, the eight domains form consistently a single stable factor and account for about 50% of the variance in Australia and other countries (21).

PWB test is a test that has been standardized and normalized by criteria and regulations of Australia. For providing the required grounds in adapting and standardizing this test in Iran, its scientific state, validity and reliability should have been qualified. Hence, the general aim of this research was introductory standardization of PWI-A in mothers of mentally retarded students of exceptional schools in north districts of Tehran and its special targets were as follows:

1. Introductory evaluation of the reliability of PWI-A in north of Tehran
2. Introductory evaluation of the validity of PWI-A in north of Tehran

Accordingly, the questions of this article that we intended to answer were as follows:

1. Is PWI-A in mothers of mentally retarded students of Tehran’s north districts reliable?
2. Is PWI-A in mothers of mentally retarded students of Tehran’s north districts valid?

## Materials and Methods

From 358 mothers of mentally retarded students in north of Tehran (districts 1, 2, 3), 200 mothers were selected by systematic random sampling. Design used in this research was survey method. In order to test the research questions, SPSS software was used for data analysis, Cronbach’s alpha coefficient was used to evaluate the test reliability and the linear multivariate regression (Enter method) was used to achieve the test validity.

After obtaining the certification for research from Tehran’s exceptional education organization, we referred to Sayyad Shirazi girl’s exceptional school and Piroozi boy’s exceptional school located in north of Tehran. During this period, eight free meetings with the mothers were held and after describing the aims of this research, they were asked to complete the questionnaires. A letter including personal well-being index together with necessary explanations was sent to mothers who have not attended the meetings. They returned the letters after completion. Demographical indicators or data collection procedure were not included in the interview but this information was obtained in a separate demographic questionnaire. All of the subjects signed a written consent form.

It should be emphasized that the aim of researchers in this study has not been measurement and evaluation of mental illness, but measurement of PWB. PWB index is derived from a quality of life questionnaire that measured seven domains and then, to make a short and international indicator, an essential factor covering all aspects was defined by quality of life control center in Australia for each domain. Test constructors have also published information in scale guide (21).

The PWB Scale developed by Cummins (21) was used in this study to measure PWB. It is generally agreed that SWB can be measured through questions of satisfaction directed to people’s feelings about themselves (21). It is based on life domain scale, in which there is a domain-level representation of global life satisfaction and 7 other domains that their scores are computed as PWB. Each item refers to a specific life domain (aspect) and the scores of all items are averaged to produce a measure of SWB. PWB scale contains seven items of satisfaction, each one corresponding to a quality of life domain including standard of living, health, achieving in life, relationships, safety, community-connectedness, and future security. These seven domains are theoretically embedded as representing the first level deconstruction of the global question: "How satisfied are you with your life as a whole?" (21). Its basic psychometric characteristics in Australia have been described (28). Sixteen surveys of the Australian population have produced a maximum variation of 3.2 percentage points in PWB. Cronbach’s alpha is reported to be between 0.70 and 0.85 in Australia and overseas. Inter-domain correlations are often moderate at around 0.30 to 0.55 and item-total correlations are at least 0.50. The index has also demonstrated good test-retest reliability across a 1-2-week interval with an intra-class correlation coefficient of 0.84 (21). A correlation of .78 with the life scale that was developed by Ed Diener and colleagues (25) has been reported (26). Scores on the satisfaction with life scale (SWLS) correlate moderately to highly with items of Personal well-being (25).

## Results

Two hundred mothers of mentally retarded students had a minimum age of 28 and a maximum age of 56 years and their mean age was 35.59 ± 18.66 . Demographic data is demonstrated in table 1.

**Table 1:** Demographic data of mentally retarded students’ mothers

Group	Students’ Sex		Age	Age
	Female	Male	Range	Mean ± SD
Mothers	100	100	28-56	35.59 ± 18.66

Table 2 shows statistical indexes including mean and standard deviations of seven items. It can be observed that minimum number is related to the seventh item with an average of 6.20 and the highest score is related to the fourth item with an average of 7.29.

**Table 2:** Descriptive statistics of seven items of Personal Well-being Index – Adult (PWI-A)

Items	Mean	SD	Normality
PWB1	6.50	2.729	0.94
PWB 2	7.04	2.741	0.96
PWB 3	6.54	2.751	0.92
PWB 4	7.29	2.860	0.88
PWB 5	6.75	3.001	0.98
PWB 6	7.08	2.867	0.82
PWB 7	6.20	2.886	0.95

*Reliability Criteria and Factor Analysis*

To measure internal consistency of PWI-A, Cronbach’s alpha coefficient was used.

Tables 3 show the results of reliability analysis. Cronbach’s alpha coefficient of PWI-A was 0.90 that is statistically acceptable for determining the reliability of the questionnaire and is above the normal range. The correlation between the seven items of PWI-A was 0.72. Besides, studying on internal consistency of these items showed that all seven items had correlation with total score and their scores averages were similar to each other. This indicates that test questions have reliability with regard to evaluation of a common feature.

**Table 3:** Results of reliability analysis of Personal Well-being Index-Adult (PWI-A

Item	Scale mean if item Deleted	Scale variance if Item Deleted	Corrected Item-Total Correlation	Alpha if Item Deleted
PWB1	40.8850	190.4339	0.7222	0.8924
PWB2	40.3550	190.6020	0.7158	0.8931
PWB3	40.8550	186.2653	0.7784	0.8863
PWB4	40.0950	188.8301	0.7032	0.8944
PWB5	40.6450	184.5015	0.7209	0.8926
PWB6	40.3150	186.8098	0.7304	0.8914
PWB7	40.1900	190.2150	0.6749	0.8976

It should be mentioned that one hundred mothers of mentally retarded students participated in test-retest reliability study. The correlation between the two administrations of PWI-A (coefficients of Cronbach’s alpha) was 0.81.

*Validity Criteria*

Table 4 shows the results of KMO and Bartlett's test. It can be observed that sample size was sufficient for analysis. Bartlett test of sphericity resulted in a Chi-square value of equal to 818.974 with 406 degrees of freedom that was meaningful in an alpha level of 0.001.

**Table 4:** Results of KMO and Bartlett's Test

Kaiser-Meyer-Olkin Measure of Sampling Adequacy	0.891
Bartlett's Test of Sphericity Approx. Chi-Square	818.974
df	21
Sig	0.001

Table 5 shows the results of factor matrix of converged varimax rotation (factors, items, factors loading, eigenvalues and variance percent).

*Construct Validity*

Since this scale has seven items and each item measures comments and feedback of the person about one area of personal well- being, the scale manufacturers believe that the individual score in each area plays an important role in the distribution of overall life satisfaction scores (The first single item and separated from the other seven items). Therefore, scale manufacturers have recommended linear multivariate regression analysis for validity assessment, in which individual score in single item of overall life satisfaction as the dependent variable and the seven items scales as prediction variables are considered.

**Table 5:** Results of factor matrix of converged varimax rotation

Component	Initial Eigenvalue			Extraction Sums of Squared Loadings		
	Tota**l**	% of Variance	Cumulative %	Total	% of Variance	Cumulative %
1	4.495	64.216	64.216	4.495	64.216	64.216
2	.703	10.045	74.261			
3	.557	7.960	82.221			
4	.397	5.676	87.897			
5	.335	4.784	92.681			
6	.278	3.968	96.649			
7	.235	3.351	100.000			

Table 6 shows the results of this regression analysis, in which the expressive "F" index is meaningful.

**Table 6:** ANOVA-Linear multivariate regression by Enter method for Tehran’s north districts

Model		Sum of Squares	df	Mean squares	F	Sig
	Regression	122070.5	7	17438.646	73.476	0.001
	Residual	45568.976	192	237.338		
	Total	167639.5	199			

After implementation of linear multivariate regression analysis with Enter method, it was determined that the scale items can predict 78% of distribution of overall life satisfaction. Besides, there was a meaningful relation between the questions of part one and the questions of part two and this scale has shown the required validity.

Therefore, regression coefficients were calculated for the seven items and the results are shown in table 7.

**Table 7:** T levels, standardized and unstandardized coefficients of seven items of PWI-A

Unstandardized Coefficients			Standardized Coefficients		
	Std.Error	B	Beta	t	Sig.
(Constant)	3.484	.478		.140	.009
PWB1	.605	5.094	.479	8.419	.001
PWB2	.585	3.133	.296	5.359	.001
PWB3	.663	-.105	-.010	-.159	.874
PWB4	.615	2.201	.220	2.327	.044
PWB5	.542	1.566	.162	2.887	.004
PWB6	.628	1.609	.160	1.969	.034
PWB7	.541	.351	.035	.649	.517

Table 7 shows that the items 1, 2, 4, 5, 6 in anticipation distribution of overall life satisfaction have a significant contribution while items 3 and 7 have no significant influence. Items 1 and 2 have the largest slope coefficients.

It can be concluded that PWI-A scale is valid among mothers of mentally retarded students of Tehran north districts and has a proper efficiency in evaluating PWB among mothers of mentally retarded children. Hence, counselors are suggested to use this indicator to identify PWB of mothers of mentally retarded students.

## Discussion

PWB is an important construct, as its low levels can lead to depression or social isolation (27,28). In this regard, identification of factors that maintain high levels of SWB such as community connectedness are important for developing strategies to prevent problems associated with low levels of SWB. Participation in voluntary activities and strategies individuals use to gain control over their life are possibly related to how satisfied individuals are about their personal life (17, 29-32). Diener, Oishi and Lucas (33) define SWB as a reflection of how people perceive and evaluate their lives and the environment around them.

Considering what was mentioned, this is the first study which evaluates validity and reliability of the PWI-A in mothers of mentally retarded students. Results showed that the seven-item PWI-A scale has a high reliability with a Cronbach’s alpha of 0.90. Our findings are consistent with the results of the previous studies performed by Lau and Cummins (21), Miller (19), Agha Yousefi (23), Davey (24) and Davern (34), reported a reliability between 0.72 and 0.90 for PWI-A.

With regard to the extracted data from the results of this research, it can be concluded that although retardation and social and psychological problems of society can create tenses in an individual and some of them can not be eliminated without elimination of the main cause, some of individual tenses result from defense system failure or tenses at mental organization level, more exactly, personality, that leads to depression, lower self- respect, self- confidence and psychomotor energy and also creates some barriers against independence, social justice and consistent development with society beliefs (35). The behaviors of a living organism originates from a general structure that is called “personality” and totality, relative persistence, dynamism, individuality and monotheism are five relatively common concepts.

Other results showed that PWI-A had the most extensive coverage of construct validity. This finding is consistent with the results of the previous studies by Diener, Emmons, Larsen, Griffin (25) and Tomas (26), reported a correlation of 0.78 between the satisfaction and life scale. Cummins (21) reported the eight domains form a single stable factor and account for about 50% of the variance in Australia and other countries.

These results can be explained in several ways: The studied mothers in this research in North of Tehran have succeeded to gain peace far away from mental distress and mental agitations and insecurities and have such quiet hearts that this mental quietness has created happiness (36). Another explanation is that these mothers knew that general life satisfaction and PWB originates from peace, thought and recognition of their existences. They live with hope and do not regret and always try to be positive and hopeful. All of these factors are explanations for similar responses to PWB scale questions and lead to validity and reliability of this scale in Tehran’s north districts. These mothers do not pay attention to the problems, get friends with others and abandon all dependencies and get rid of mental distress. Consequently, they succeed to achieve peace and internal security. Another explanation is that these mothers have accepted the facts to keep family strength and respects (36). All these factors contribute to their physical hygiene improvement and better mental health and indicate that questions of PWI-A among mothers of mentally retarded students are valid and reliable.

Finally, it can be concluded that PWI-A of mothers of mentally retarded students of Tehran north districts has the required reliability and validity. Thus, we suggest the experts of exceptional education, psychology clinics and well-being organizations to use this index to identify the well-being of mothers of mentally retarded students.

*Limitations*

As a limited group of mothers of mentally retarded students were enrolled in this study, these findings can not be generalized to the whole population.

## Authors’ Contributions

ARA conceived and designed the evaluation, collected the clinical data, interpreted them, performed parts of the statistical analysis and drafted the manuscript. AA and NS participated in designing the evaluation, data collection and analysis. All authors read and approved the final manuscript.

## Notes

**Declaration of Interest:** None.
